# Insights into Asparaginase from Endophytic Fungus *Lasiodiplodia theobromae*: Purification, Characterization and Antileukemic Activity

**DOI:** 10.3390/ijerph19020680

**Published:** 2022-01-07

**Authors:** Hani A. Moubasher, Bassem A. Balbool, Yosra A. Helmy, Amnah Mohammed Alsuhaibani, Ahmed A. Atta, Donia H. Sheir, Ahmed M. Abdel-Azeem

**Affiliations:** 1 Botany and Microbiology Department, Faculty of Science, Cairo University, Giza 12613, Egypt; moubasher@sci.cu.edu.eg; 2 Biotechnology Department, Faculty of Biotechnology, October University for Modern Sciences and Arts, 6th October 12451, Egypt; 3 Department of Animal Hygiene, Zoonoses and Animal Ethology, Faculty of Veterinary Medicine, Suez Canal University, Ismailia 41522, Egypt; yosra_helmy@vet.suez.edu.eg; 4 Department of Physical Sport Science, College of Education, Princess Nourah bint Abdulrahman University, P.O. Box 84428, Riyadh 11671, Saudi Arabia; amalsuhaibani@pnu.edu.sa; 5 Department of Physics, College of Science, Taif University, P.O. Box 11099, Taif 21944, Saudi Arabia; a.atta@tu.edu.sa; 6 Chemistry of Natural and Microbial Products Department, National Research Center, Giza 12622, Egypt; donia_sheir@yahoo.com; 7 Botany and Microbiology Department, Faculty of Science, Suez Canal University, Ismailia 41522, Egypt

**Keywords:** metabolites_1_, endophytic fungi, asparaginase, induction, purification, antileukemia

## Abstract

Endobiotic fungi are considered as a reservoir of numerous active metabolites. Asparaginase is used as an antileukemic drug specially to treat acute lymphoblastic leukaemia. The presented study aims to optimize the media conditions, purify, characterize, and test the antileukemic activity of the asparaginase induced from *Lasiodiplodia theobromae*. The culture medium was optimized using an experiment designed by The Taguchi model with an activity ranging from 10 to 175 IU/mL. Asparaginase was induced with an activity of 315 IU/mL. Asparaginase was purified with a specific activity of 468.03 U/mg and total activity of 84.4 IU/mL. The purified asparaginase showed an approximate size of 70 kDa. The purified asparaginase showed an optimum temperature of 37 °C and an optimum pH of 6. SDS reduced the activity of asparaginase to 0.65 U/mL while the used ionic surfactants enhanced the enzyme activity up to 151.92 IU/mL. The purified asparaginase showed a K_m_ of 9.37 µM and V_max_ of 127.00 µM/mL/min. The purified asparaginase showed an IC_50_ of 35.2 ± 0.7 IU/mL with leukemic M-NFS-60 cell lines and CC_50_ of 79.4 ± 1.9 IU/mL with the normal WI-38 cell line. The presented study suggests the use of endophytic fungi as a sustainable source for metabolites such as asparaginase, provides an opportunity to develop a facile, eco-friendly, cost-effective, and rapid synthesis of antileukemic drugs, which have the potential to be used as alternative and reliable sources for potent anticancer agents.

## 1. Introduction

Endobiotic (endophytic) fungi are microfungi that live inside the host plant tissue intercellularly and/or intracellularly without any apparent pathological symptoms [1]. To be able to sustain steady symbiosis, endophytes secrete chemical substances to help the plants to acclimatize better to the harsh environment [2]. As a treasure mine of bioactive metabolites, endophytic fungi are a sustainable source of various natural products *viz:* quinones, saponins, alkaloids, steroids, phenolic acids, terpenoids, and tannins that exhibit antimicrobial and anticancer properties [3].

Asparaginase (EC 3.5.1.1) is classified among amidohydrolases, it hydrolyzes the asparagine into aspartic acid (aspartate) and ammonium (NH_4_^+^) [4]. Clementi [5] reported asparaginase for the first time in the serum of guinea pig. Furthermore, Mashburn [6] extracted asparaginase from *E.coli* and reported its antileukemic properties for the first time.

Clinical data published over the last two decades suggests that asparaginase is a vital element in the treatment of acute lymphoblastic leukaemia (ALL) [6,7]. Both normal and leukemic cells require the amino acid asparagine for the proliferation process. Normal cells can synthesize asparagine for their growth with the help of asparagine synthetase (AS, EC 6.3.5.4). While, neoplastic cells lack asparagine synthetase [8], thus they are dependent on the serum asparagine to grow and proliferate. The administration of asparaginase to ALL patients depletes the serum asparagine due to its hydrolytic activity, which will lead to inhibition of neoplastic cells proliferation process and in return it will die off and arrest in G-1 phase [9].

The extracellular production of asparaginases is mainly dependent on the optimization of cultural conditions, to produce it in a bulk amount with a cheap method. The production is mainly based on optimizing the carbon and nitrogen sources, other parameters as; pH, temperature and inoculum size [10].

Different methods were reported to produce asparaginase from microorganisms including solid state fermentation and submerged fermentation—induction and purification methods vary between microorganisms [7,8,9,10]. Several trials were designed by scientists to purify asparaginase with a high purity and yield in order to use asparaginase as a drug [11,12,13,14]. The trials included the precipitation of total protein using ammonium sulphate followed by ion exchange column chromatography [15].Other reports used ammonium sulphate followed by size exclusion gel filtration chromatography [16,17]. Other researchers used the affinity columns for direct purification of asparaginase [18].

Purified asparaginase from *E. coli* and *Erwinia chrysanthemi* was used clinically to treat the acute lymphoblastic leukemia, but it showed allergic side effects which led to death [19,20,21]. The harsh side effects associated with the bacterial asparaginase was related to the prokaryotic origin of the purified enzyme [19]. Hence, researchers started to target fungi as a safe alternative eukaryotic source to produce asparaginase to decrease the associated allergic reactions [22]. Asparaginases were recorded from several fungal species such as *Mucor hiemalis*, *Aspergillus niger*, *A. flavus*, *A. nidulans*, *A. terreus* [23,24].

Due to the ability of endophytes to produce several enzymes and other natural products with high activity, researchers targeted endophytic fungi as a source of asparaginase. Balbool et al. screened the ability of 25 endophytic fungal isolates to produce asparaginase, 7 isolates showed positive asparaginase results. *Lasiodiplodia theobromae* recorded the highest asparaginase activity out of the tested isolates [24].

The present study is focusing on the production of asparaginase with maximum activity by optimizing the medium condition and induction of asparaginase from *Lasidiplodia theobromae*, followed by the purification of asparaginase and the characterization of the purified asparaginase. Furthermore, the antileukemic activity of the purified asparaginase was tested against the mouse myelogenous leukemia carcinoma cells (M-NFS-60).

## 2. Materials and Methods

### 2.1. Microorganism

*Lasiodiplodia theobromae* was obtained from the Suez Canal University Fungarium, Ismailia, Egypt (SCUF-TP2016). It was selected based on a continuing work from previous studies carried out by Balbool [24].

### 2.2. Optimization of Media Conditions and Asparaginase Induction

To optimize the culture conditions to produce asparaginase with maximum activity, a Taguchi statistical model was used [25]. The experimental design created several conditions as orthogonal arrays to ensure an efficient and reproducible experiment with minimal errors. During the presented experimental design three variables were studied under temperature of 28 °C; asparagine (nitrogen source), glucose (carbon source), and pH. Each of the three variables is represented by three variation levels. The experiment size was nine trials (layout L9) with a degree of freedom 8 [26], (for more details please check supplementary materials).

To produce asparaginase with maximum activity; 5 mm disks of *L. theobromae* were inoculated in 10 flasks containing 100 mL Malt Extract Agar media (MEA) and incubated for 7 days at 28 °C. One liter of 1% asparagine (substrate) was prepared in bi distilled water and distributed into 10 flasks (100 mL), and then autoclaved at 121 °C for 15 min. Grown fungal mats (average 0.2 g for each flask) were transferred into each flask and incubated at 28 °C for 48 h.

Enzyme activity was estimated with directed Nesslerization according to Wriston [27] using asparagine as substrate. The enzyme activity was expressed as international unit (IU); IU is defined as the amount of enzyme needed to liberate one micromole of ammonium per minute at pH = 7.00 and 37 °C. The activity was estimated and compared to the results out of Taguchi design.

### 2.3. Purification of Asparaginase

#### 2.3.1. Total Protein Precipitation

The extracellular protein precipitation was carried out by cold acetone precipitation method [28]. Acetone was kept at −80 °C prior to precipitation, 2× cold acetone was added to the culture filtrate and incubated at −20 °C for 2 h. The samples were centrifuged at 13,000 rpm for 15 min at 4 °C. The supernatant was discarded, and the pellet obtained were dissolved in a suitable volume of 50 mM Tris buffer (pH 7) and stored at −80 °C till further use.

#### 2.3.2. Ion Exchange Chromatography

The collected precipitate of asparaginase was further fractionated by prepacked ion exchange column (Hi-trap-Q-FF, 5 mL, GE). The column was equilibrated by 5× of column volume of Tris buffer (till reaching UV baseline stability) (50 mM, pH 7). The enzyme was eluted by gradient concentrations of NaCl (0–1 M) with a rate of 1.5 mL/min. The activity was estimated. The fractions that showed high enzymatic activity was collected together, concentrated using chilled acetone and the enzyme activity and total protein were estimated. The collected fractions were further purified using gel filtration chromatography using Sephadex G-100.

#### 2.3.3. Size Exclusion Gel-Filtration Chromatography

The collected precipitate of asparaginase from the ion exchange column was further fractionated by gel- filtration Sephadex G-100 column. After 2 days swelling of Sephadex G100 particles, the resin was poured to the column (2 × 40 cm), and the column was equilibrated by Tris buffer (50 mM, pH 7), with a flow rate of 12 mL/h. The enzyme was loaded to the top of the column, eluted by the same buffer, and the activity, protein contents, and molecular homogeneity of the eluted fractions were assessed. The most active fractions collected together to be checked on SDS–PAGE.

### 2.4. Estimation of Total Protein

Total protein concentration was estimated by measuring the absorbance at 280 nm. First, the standard curve of Bovine serum albumin (BSA) was created. Different dilutions of BSA ranging from 50 to 450 µg/mL were prepared by adding the adequate volume of BSA stock solution (1 mg/mL) and double distilled water in test tubes. The absorbance versus concentration graph was used to estimate the protein content of the unknown protein sample (Supplementary Materials).

#### Estimation of Enzyme Molecular Weight Using SDS PAGE

The homogeneity of the bands and the molecular weight of the purified asparaginase were estimated using SDS-PAGE according to the instructions of Laemmli [29], using 12% separating gel (6.8) and 5% stacking gel (pH 8.8). The molecular weight of the appeared protein bands was calculated from the inference of authentic protein marker (BLUEstain™ 2 Protein ladder, 5-245 kDa). The marker consists of approximately 0.1–0.4 mg/mL of each protein in the buffer (20 mM Tris- phosphate, pH 7.5 at 25 °C), 2% SDS, 0.2 mM Dithiothreitol, 3.6 M Urea, and 15% (*v*/*v*) Glycerol). The protein bands were stained with Coomassie brilliant blue R-250.

### 2.5. Enzyme Characterization

#### 2.5.1. Determination of the Optimum Reaction Temperature and Stability

To study the effect of temperature on the purified asparaginase, 0.25 mL of the purified enzyme was pre-incubated with 0.75 mL of 50 mM Tris buffer (pH 7) at temperature range from 20 °C to 70 °C. Followed with assaying the asparaginase activity under the standard reaction conditions [30,31]. The temperature stability was studied by mixing 1.5 mL of purified enzyme and 4.5 mL buffer (pH 7) together and incubated at 37 °C. Removed each time at different time intervals (0 min, 30 min, 1 h, 2 h, 4 h, 5 h and 24 h) was 1 mL of the mixture, and the enzyme activity was estimated according to Mashburn and Wriston [6].

#### 2.5.2. Effect of pH on Asparaginase Activity

The effect of pH on asparaginase activity was studied over a pH range of 3 to 10 using different buffers; acetate buffer (pH 3, 4 and 5), Phosphate buffer (pH 6–6.5), and Tris base buffer (pH 7–10) [11]. The assay was performed by pre-incubating 0.25 mL of purified enzyme with 0.75 mL of each of the buffers at 37 °C for 30 min. The enzyme activity was estimated according to Mashburn and Wriston [6].

#### 2.5.3. Effect of Inhibitors, Activators on Asparaginase Activity

The influence of inhibitors and activators (2 mM) was analyzed using ethylenediaminetetraacetate (EDTA), N-ethylemaleimide (NEM), phenylmethylsulphonylfluoride (PMSF), Triton-X 100, Tween-80, sodium dodecyl sulphate (SDS), and β-mercaptoethanol (β-ME) [32]. A pre-incubation was conducted for 30 min after mixing 0.25 mL of the enzyme with 0.75 mL of the concerned inhibitor/activator. The activity was determined as previously mentioned.

#### 2.5.4. Enzyme Kinetics

The kinetic parameters (K_m_ and V_max_) of the purified asparaginase were determined by linear regression from Lineweaver-Burk and Michaelis-Menten plots with different concentrations of asparagine (substrate) (0 mM–20 mM) [11].

### 2.6. Testing the Antileukemic Activity

#### Cytotoxicity Evaluation Using the Viability Assay

*Cell line propagation*

WI-38 cells (human lung fibroblast normal cells) were propagated in Dulbecco’s modified Eagle’s medium (DMEM) supplemented with 10% heat-inactivated fetal bovine serum, 50 µg/mL gentamycin, 1% L-glutamine and 4-(2-hydroxyethyl)-1-piperazinethanesulfonic acid (HEPES) buffer. All cells were maintained at 37 °C in humidified atmosphere with 5% CO_2_ and were sub-cultured two times a week [33].

M-NFS-60 (mouse myelogenous leukemia carcinoma cells) were propagated in RPMI-1640, supplemented with 10% heat-inactivated fetal bovine serum, 1% L-glutamine, HEPES buffer, and 50 µg/mL gentamycin. All cells were maintained at 37 °C in a humidified atmosphere with 5% CO_2_ and were sub-cultured two times a week [34].

*Cytotoxicity evaluation*

Cytotoxicity of the purified asparaginase was tested on mouse leukemia cell line: M-NFS-60 (mouse myelogenous leukemia carcinoma cells) to determine the Median Minimal Inhibitory Concentration (MIC_50_) and the mammalian cell line: WI-38 cells (human lung fibroblast normal cells) to verify the Median Minimal Cytotoxic Concentration (MCC_50_), according to methods described by [35]. Cells were seeded in 96-well plate at a cell concentration of 1 × 10^4^ cells per well in 100 µL of growth medium. Fresh medium containing serial two-fold dilutions of the purified asparaginase were prepared. After 24 h of seeding, the media containing serial asparaginase were added to confluent monolayer cells dispended into 96-well, flat bottomed micro-titer plates (Falcon, NJ, USA) using multichannel pipette. Three wells were used for each concentration of the test sample (experiments were carried in triplicates), control cells were incubated without test sample. The micro-titer plates were incubated at 37 °C in humidified incubator with 5% CO_2_ for a period of 24 h. After the end of incubation period, media were aspirated, and 1% crystal violet solution was added to each well for 30 min. The stain was removed, and the plates were rinsed using tap water until all excess stain is removed. Glacial acetic acid (30%) was then added to all wells and mixed thoroughly, and then the absorbance of the plates were measured after gently shaken using microplate reader (Tecan Trading AG, Switzerland.) at a wavelength of 490 nm. All the results were corrected for background absorbance detected in wells without adding stain. Treated samples were compared with the cell control in the absence of the purified asparaginase.

Cell cytotoxicity effect of the purified asparaginase was calculated to determine the number of viable cells and the percentage of viability was calculated as.

$$\mathrm{Viability}\;\mathrm{percentage}=\frac{\mathrm{ODt}}{\mathrm{ODc}}\times 100$$

where ODt is the mean optical density of the three wells treated with each concentration of asparaginase, ODc is the mean optical density of untreated cells.

The relation between the surviving cells and the asparaginase used concentrations was plotted to get the survival curve of each tumor cell line after treatment with the purified asparaginase. The cytotoxic concentration (MCC_50_), the concentration required to cause toxic effect in 50% of intact cells, was estimated from graphic plots of the dose response curve for each concentration, using prism software (San Diego, CA, USA).

### 2.7. Statistics

The data were presented in tables using the Microsoft excel 365, figures were created using the Prism ver. 8. All experiments were carried out in triplicates, all data were reported with mean ± SEM.

## 3. Results

### 3.1. Optimization of Media Conditions

**Figure 1:**
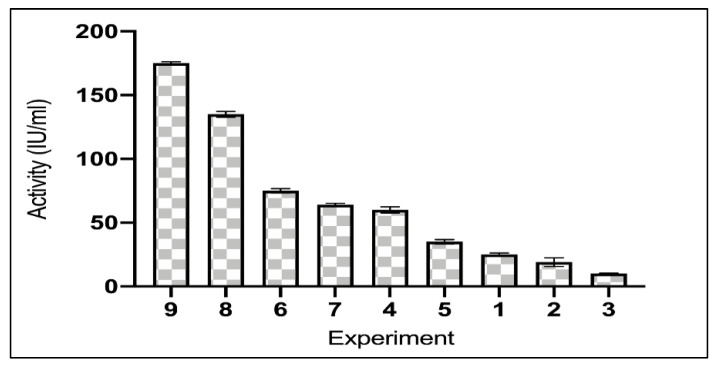
Asparaginase activities of Taguchi design experiments.

Among the nine different experiments that were performed according to Taguchi model, two of them; experiment 9 and 8 showed a high asparaginase activity with activities of 175 IU/mL, 135 IU/mL respectively, other experiments showed activities ranging from 75–10 IU/mL (Figure 1). However, on inducing asparaginase the enzyme activity was estimated to be 315 IU/mL.

### 3.2. Purification of Asparaginase

**Figure 2:**
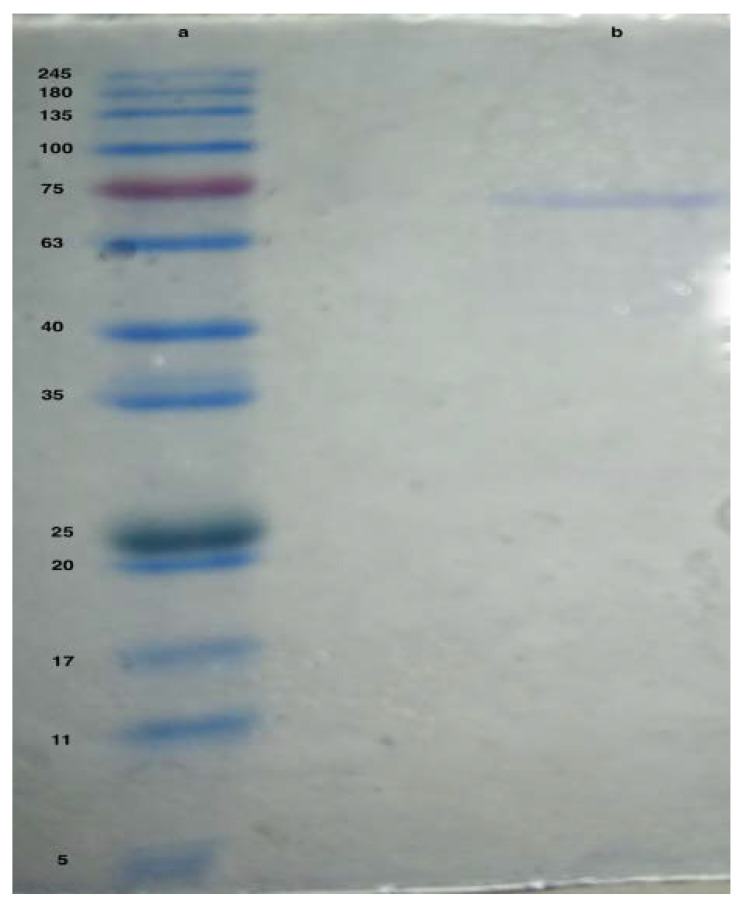
Purified asparaginase from *L. theobromae* on SDS-PAGE. (**a**) protein ladder, (**b**) purified asparaginase.

The purification of asparaginase from *L. theobromae* was carried out with a combination of some steps, starting with total protein precipitation using chilled acetone, ion-exchange chromatography on Q-FF column, and gel filtration Sephadex G-100. After precipitation of the induced enzyme with chilled acetone, asparaginase showed a specific activity of 36.71 IU/mg with a total activity of 250 IU/mL in a 6.81 mg/mL total protein (Table 1). Concentrated enzyme was loaded into pre-equilibrated Q-FF ion exchange column and eluted using gradient concentrations of NaCl (0–1 M). All fractions eluted only with 0.3 M NaCl showed asparaginase activity (Figure 2). All fractions showed high asparaginase activity were collected together and concentrated, the asparaginase activity was estimated to be 138.91 IU/mL and the specific activity was 105.88 U/mg. For further purification, concentrated fractions were loaded into a pre-equilibrated Sephadex G-100 column. All positive fractions were collected together and concentrated. Collected fraction showed a total activity of 84.4 IU/mL, a specific activity of 468.03 U/mg compared to the induced enzyme specific activity (Table 1) (for more details please check supplementary materials).

#### Estimation of Enzyme Molecular Weight

The purified asparaginase showed a band around 70 kDa compared to the reference protein ladder (Figure 2).

### 3.3. Enzyme Characterization

#### 3.3.1. Effect of pH on Asparaginase Activity

**Figure 3:**
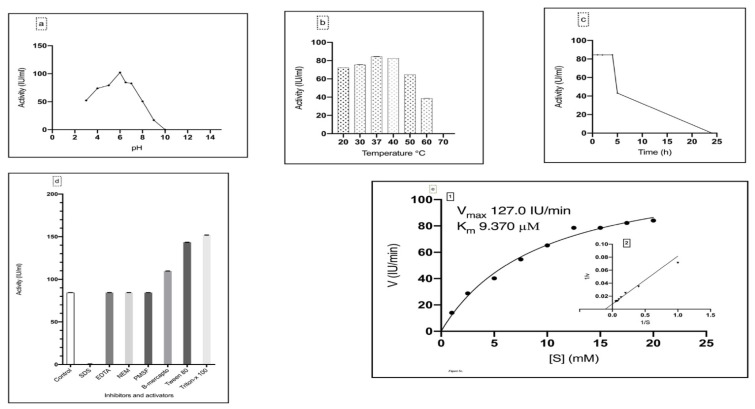
(**a**) The effect of pH on the purified asparaginase from *L. theobromae*. (**b**) The effect of temperature on the purified asparaginase from *L. theobromae*. (**c**) Thermal stability of purified asparaginase from *L. theobromae* at 37 °C. (**d**) Effect of inhibitors and activators on purified asparaginase from *L. theobromae*. (**e**) Purified asparaginase kinetics. 1. Michaelis Menten plot for the purified asparaginase enzyme from *L. theobromae*. 2. The different activities value of purified asparaginase from *L. theobromae* with increasing the substrate concentrations.

According to the results presented in Figure 3a, the optimum pH for the purified asparaginase was 6, while at pH 10 the enzyme lost its activity, starting from pH 3 the enzyme retained 51.22% of its activity, while at pH 4, 5 retained 72.22%, 77.43% respectively of its activity, the enzyme activity started to decrease simultaneously with increasing the pH value.

#### 3.3.2. Effect of Temperature on Asparaginase Activity

The purified asparaginase showed an activity over a wide range of temperatures (20–60 °C). The optimum temperature was 37 °C with an activity of 84.41 IU/mL, followed by 40 °C with 82.52 IU/mL (Figure 3b).

The purified asparaginase showed complete stability at 37 °C for 1, 2 and 4 h, while the activity decreased to 43.2 IU/mL after 5 h. The enzyme lost its activity totally after 24 h (Figure 3c).

#### 3.3.3. Effect of Inhibitors and Activators on Asparaginase Activity

The effect of different inhibitors and activators was studied on the purified asparaginase, where SDS showed a reduction in the enzyme activity to 0.65 IU/mL (10.35% relative activity), while EDTA, NEM, and PMSF showed no effect on asparaginase activity, in contrast β-mercaptoethanol, Tween 80, and Triton-x 100 enhanced the enzyme specific activity to 130%, 143.48% and 151.92% respectively regarding the control (Figure 3d).

#### 3.3.4. Determination of Enzyme Kinetics

According to results in Figure 3e 1, 2, Michaelis constant K_m_ and maximum velocity V_max_ of the purified asparaginase from *L. theobromae* were 9.370 µM and 127.00 IU min^−1^ respectively.

### 3.4. Estimation of Asparaginase Antileukemic Activity

#### 3.4.1. Evaluation of Cytotoxicity against M-NFS-60 Cell Line

**Figure 4:**
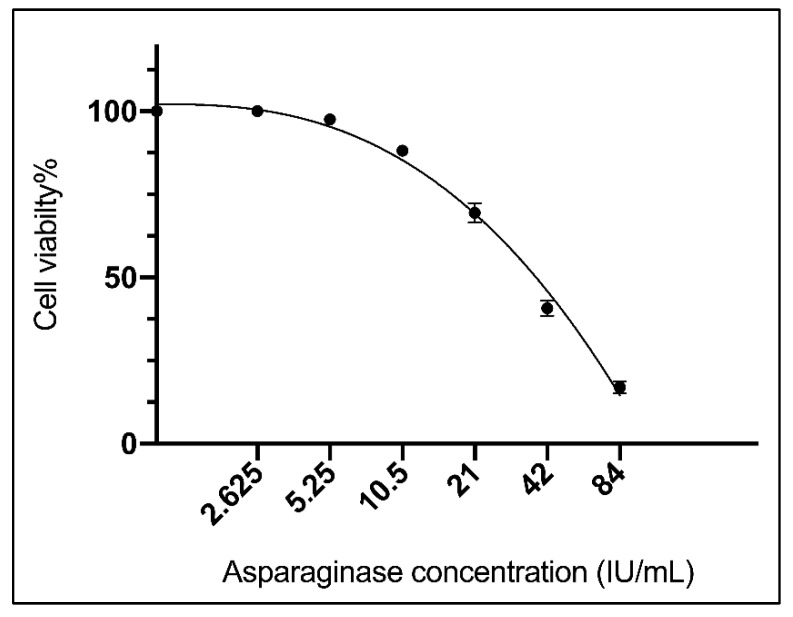
Viability response of M-NFS-60 cell line against serial two-fold dilutions of purified asparaginase from *L. theobromae*.

Different concentrations of the purified asparaginase starting with 84.00 IU/mL to 2.62 IU/mL were tested against mouse myelogenous leukemia carcinoma cells to estimate the MIC_50_ value, results revealed that the MIC_50_ was 35.2 ± 0.7 IU/mL (Figure 4).

#### 3.4.2. Evaluation of Cytotoxicity against WI-38 Cell Line

**Figure 5:**
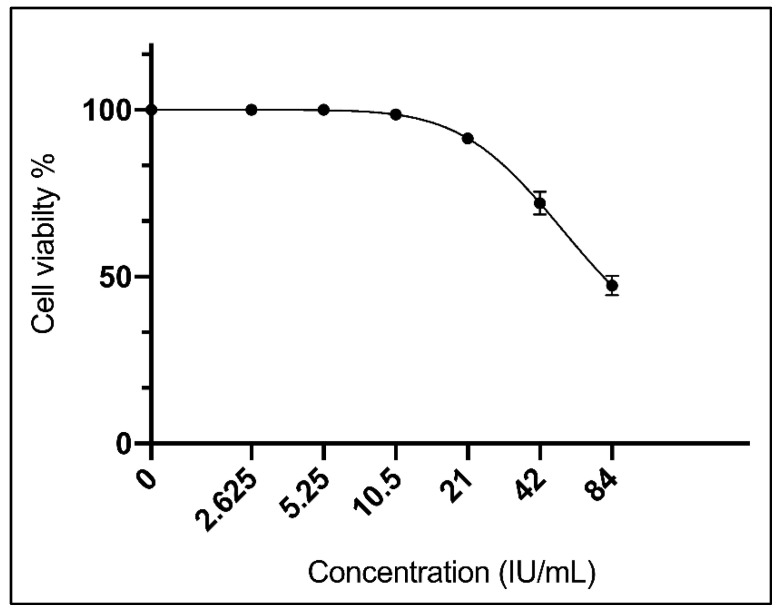
Viability response of WI-38 cell line against serial two-fold dilutions of purified asparaginase from *L. theobromae*.

Different concentrations of the purified asparaginase starting with 84.00 IU/mL to 2.62 IU/mL were tested against WI-38 cells (human lung fibroblast normal cells) to estimate MCC_50_ value, results revealed that the MCC_50_ was 79.4 ± 1.9 IU/mL (Figure 5).

## 4. Discussion

Zhao et al. [36] and Jia et al. [37] reported that the activity of the bioactive metabolites produced by endophytes is considered similar or higher than those produced by host plants. Schulz et al. [38] reported that plant defense mechanisms and fungal virulence factors develop a balance mechanism by secreting different novel bioactive metabolites.

*Lasiodiplodia theobromae* was reported to produce several bioactive compounds used as anticancer, antifungal, antioxidant, anti-inflammatory such as: Lasiodiplodin [39], amylase [40], Paclitaxel [41], Felix [42] reported L. theobromae strain with the ability to produce: (3*S*,4*S*)-4-acetyl-3-methyl-2-dihydrofuranone, (2*R*/2*S*,3*S*,4*S*)-3-epibotryodiplodin, and jasmonic acid.

Our results showed that L. theobromae produces asparaginase extracellularly with a high enzymatic activity, while, Narayana et al. [43] reported that most of the asparaginase with microbial sources is intracellular. Extracellular asparaginase interacts minimally with the cellular constituents and is easier to extract; hence, the induction of extracellular asparaginases with high activity would be more advantageous.

To produce asparaginase with maximum activity, nine experiments were designed using Taguchi model. Taguchi model results showed a variation of asparaginase activity from 175 to 10 IU/mL, the variation of the results obtained were compatible with Ali et al. [34] who designed a model of 19 experiments for the optimization of asparaginase from *A. sydowii* and *F. oxysporum* with asparaginase activity ranging from 37-146 IU/mL and 33 to 143 IU/mL respectively. Ashok et al. [41] used Taguchi design to simulate nine experiments with asparaginase activities ranging from 3.94 to 20.57 IU/mL from *Trichosporon* asahii.

Another experiment designed for the induction of asparaginase with a maximum activity by incubating a five-days-old culture mat of *L. theobromae* with asparagine for two days and the asparaginase activity was estimated as 315 IU/mL. The incubation of the grown *L. theobromae* mat with asparagine solely as a substrate for asparaginase enhanced the selective release of asparaginase with a maximum activity with other extracellular enzymes, Nossal et al. [44] designed a similar experiment for the selective release of some degrading enzymes viz: alkaline phosphatase, cyclic phosphodiesterase, 5’-nucleotidase, acid phosphatase from E.coli by osmotic shock using different salts concentrations.

Asparaginase from *L. theobromae* was purified with a total activity of 84.40 IU/mL, specific activity of 468.03 IU/mg, and 13.68 purification folds. The estimated activity is considered low compared to Thakur et al. [11] who reported an activity of 222.18 IU/mL and a specific activity of 69.43 IU/mg, Mohamed et al. [45] reported an activity of 127 IU/mL and a specific activity of 846.IU/mg, and it was higher than the activity reported by Abbas [46] who reported an activity of 13.1 IU/mL and 0.4 IU/mg specific activity. The variation of activities would be related to the growth condition of each organism, the variation of extraction protocols.

To estimate the molecular weight of the purified asparaginase using SDS-PAGE, it was compared against known protein marker. SDS-PAGE showed a band of asparaginase by around 70 kDa. The presented result is similar to Huang [47] who reported a weight of 72 kDa and Hong [48] who reported an asparaginase of 71 kDa. Asparaginases showed a variation on the molecular weights where, Cedar and Schwartz [49] and Shifrin et al. [50] reported asparaginase from *E. coli* with 141 kDa, Scheetz et al. [51] reported asparaginase from *Fusarium tricinctum* with 161–170 kDa, Manna et al. [52] reported asparaginase from *Pseudomonas stutzeri* with 34 kDa and El-Bessouny [13] who recorded asparaginase from *Pseudomonas aeruginosa* at 160 kDa.

The purified asparaginase was characterized, where it showed a working pH range of 3–9, with an optimum pH of 6; Abbas [46] reported a working pH of 3–12 and an optimum pH of 10, on the other hand, Thakur [11] reported a working pH range of 3–10 and optimum pH of 7.

On testing the working temperature range, purified asparaginase showed activity over a temperature range of 20–60 °C with an optimum temperature of 37 °C which is in agreement with [11,45,53]. On the other hand, Gaffar and Shethna [54] reported an asparaginase isolated from *Azotobacter vinelandii* with optimum temperature of 48 °C and Mesas [55] reported an optimum temperature of 40 °C for asparaginase isolated from *Corynebacterium glutamicum*.

During the presented study some inhibitors and activators were studied against the purified asparaginase. SDS was the only reagent that showed reduction in the enzyme activity and retained 0.65% of the relative activity which was reported earlier by Thakur [11]. Which would be related to the SDS mode of action. Where it disturbs the structure of the protein which brings the folded protein structure into a linear model and coats the protein with uniform negative charges; leading to masking of the protein charges.

EDTA showed no effect on the purified enzyme which elucidated the fact that asparaginase is not an metalloenzyme, which is similar to data on *Candida utilits* by Sakamoto [56]. Regardless the fact of the non-metalloenzyme nature of the purified asparaginase some reports suggested that asparaginase is a metal activated enzyme where, El-Naggar et al. [8] reported that asparaginase from *Sterptomyces brollosae* showed an increase on activity in the presence of Mg^2+^, Mn^2+^ and Co^2+^ ions. Sonaimuthu [57] showed a metal activating characters with Mn^2+^ with asparaginase purified from *Fusarium culmorum*.

Tween 80 and Triton X-100 enhanced the activity with a relative activity of 170% and 180%, β-mercaptoethanol enhanced the enzyme activity with a relative activity of 130% our result was in agreement with Raha et al. [58]. The native protein structure mainly stabilized by the formation of disulfide bond [59], β-mercaptoethanol is a strong reducing agent that inhibit the formation of disulfide bonds in cysteine residues [60]. L-asparaginase was activated by the addition of β-mercaptoethanol. Hamza and Engel [61] reported the presence of free sulphydryl groups in the enzymes, such as cysteine residues will make it unstable and will aggregate the enzymes and the addition of β-mercaptoethanol and other ionic surfactants *viz;* Tween 80 and Triton X-100 will enhance the enzymatic activity.

NEM and PMSF showed an insignificant effect on the enzyme activity; which may be due to the absence of cysteine or serine on the active site of the purified asparaginase, our results are compatible with Thakur [11].

The activity of the purified asparaginase was assayed against different concentrations of asparagine (0–20 mM) to determine the kinetic properties, the purified enzyme showed a very low K_m_ of 9.37 µM and V_max_ of 127.00 U min^−1^. The recorded K_m_ value considered lower than the recorded K_m_ of *Mucor hiemalis*; 4 mM [11], *Penicillium*; 4 mM [62], such low K_m_ value reflects the high affinity of the purified asparaginase towards asparagine as substrate.

The anti-leukemic effect of the purified asparaginase was studied against mouse myelogenous leukemia carcinoma cells to estimate the median minimal inhibitory concentration (MIC_50_). Results revealed that the MIC_50_ was 35.2 ± 0.7 U/mL which is lower than the MIC_50_ of *A. sydowii* (50 U/mL) and *F. oxysporum* (62.5 U/mL) [31]. It is also considered higher than the MIC_50_ of *Phaseolus vulgaris* (0.75 U/mg) [45]. The lower the MIC value, the lower the doses needed and the better the efficacy of the anticancer drug [63].

Tanfous et al. [64] related the antileukemic activity of asparaginase to its capability to hydrolyze asparagine in the serum, thus depleting serum asparagine; which is essential for the proliferation of leukemic cells. Several trials were carried out by scientists to discover asparaginase and test its role in the inhibition of leukemic cells [5,65,66,67]. Asparagine is produced by asparagine synthetase (ASNS), which is encoded by ASNS gene [68]. Gervasini and Vgace [69] hypothesized that ASNS genes in the malignant lymphoblasts cells have low expression or cannot regulate the expression of ASNS when exposed to asparaginase. Thus, we can hypothesize that the leukemic cells are more dependent on the extracellular sources of asparagine, thus the administration of asparaginase will selectively depletes serum asparagine leading to suppression of the growth of leukemic cells.

The presented results showed that the MIC_50_ (35.2 ± 0.7 IU/mL) of the purified asparaginase was around double the MCC_50_ (79.4 ± 1.9 IU/mL) where MCC_50_ value represents half the cytotoxic concentration of the tested asparaginase on normal cells. Such ratio indicates that the tested asparaginase is considered safe where it requires double of the asparaginase concentration to reach the MCC_50_. With treating the leukemic cell line with the purified asparaginase, our results revealed that the carcinoma cell line reached cell death up to 83.05% with the highest concentration of the purified enzyme (84 IU/mL) which is equal to about double the MIC_50_; respectively, leukemic cells require a high concentration of asparagine to keep viable [64].

## 5. Conclusions

In conclusion, the presented results proved that endophytic mycobiota especially isolated from extreme environment is considered as a source of safe novel bioactive compounds. This study sheds the light on a new application of endophytic native taxa and it recommends the production of the extracellular induced endophytic asparaginase on a high scale to be used as a safe antileukemic drug and in food industries. Future studies must focus on the screening for bioactive compounds secreted by Egyptian endophytic mycobiota to find a safe alternative for the traditional pharmaceutical compounds or discovering a novel compound to be used in industrial, agricultural, and medical applications.

## Figures and Tables

**Table 1:** Summary of the steps involved in the purification of asparaginase from *L. theobromae*.

Step	Total Protein (mg)	Activity * (IU/mL)	Specific Activity (IUmg^−1^)	Purification Fold	Yield %
Induced enzyme	9.21	315.00	34.20	1.00	100.00
Acetone	6.81	250.00	36.71	1.07	79.37
Q-FF (0.3M NaCl)	1.31	138.91	105.88	3.10	44.10
Sephadex G-100	0.18	84.40	468.03	13.68	26.79

* One unit of asparaginase (IU) is defined as the amount of enzyme that liberates 1 μmol of ammonia min^−1^ AT 37 °C.

## Data Availability

Not Applicable

## Supplementary Materials

The following are available online at https://www.mdpi.com/article/10.3390/ijerph19020680/s1, Figure S1. Standard curve of different concentrations of BSA, Figure S2. Elution profile of asparaginase from L. theobromae from the Q-FF ion exchange column. The column was pre-equilibred with 50 mM Tris buffer (pH 7) and fractions were eluted with a gradient solution of NaCl (0-1M) prepared in same buffer, and Figure S3. Elution profile of asparaginase from L. theobromae from the gel-filtration column (Sephadex G-100). Fractions were eluted with 50 mM Tris buffer (pH 7). Table S1. The nine models generated by Taguchi design.
