# Supplementary material for: Insights into Asparaginase from Endophytic Fungus Lasiodiplodia theobromae: Purification, Characterization and Antileukemic Activity
Source: Int J Environ Res Public Health. 2022 Jan 7;19(2):680. doi: 10.3390/ijerph19020680 (PMC8775487; doi:10.3390/ijerph19020680)
Supplement: Supplementary file 1 [file ijerph-19-00680-s001.zip › ijerph-1479702-supplementary.pdf]

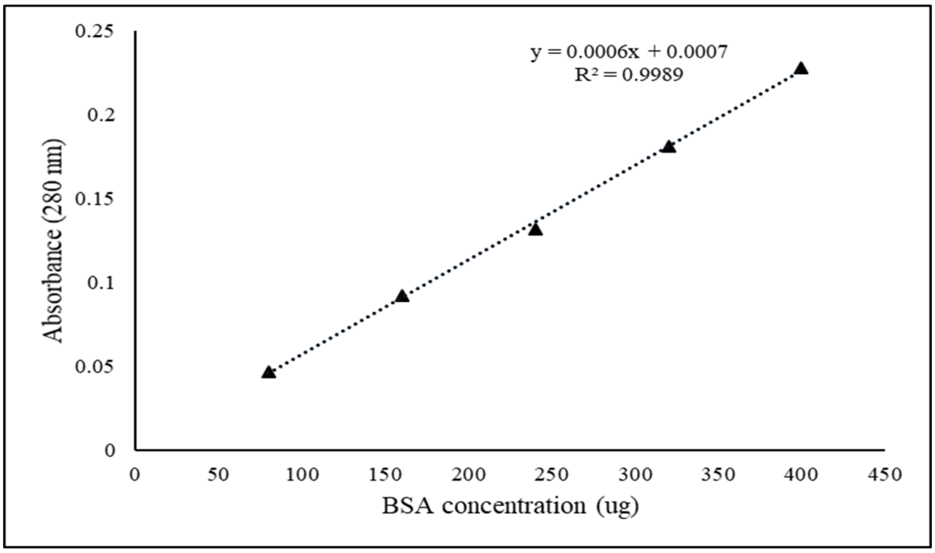

Figure S1. Standard curve of different concentrations of BSA.

Table S1. The nine models generated by Taguchi design

Exp.	*Asparagine (g/L)*	*Glucose (g/L)*	*pH*	*KH2PO4 (g/L)*	*KCl (g/L)*	*MgSO4 (g/L)*	*RPM*
1	*2.5*	*1*	*5*	*1*	*0.5*	*0.5*	*120*
2	2.5	1.5	7	1	0.5	0.5	120
3	2.5	2	9	1	0.5	0.5	120
4	5	1	7	1	0.5	0.5	120
5	5	1.5	9	1	0.5	0.5	120
6	5	2	5	1	0.5	0.5	120
7	10	1	9	1	0.5	0.5	120
8	10	1.5	5	1	0.5	0.5	120
9	10	2	7	1	0.5	0.5	120

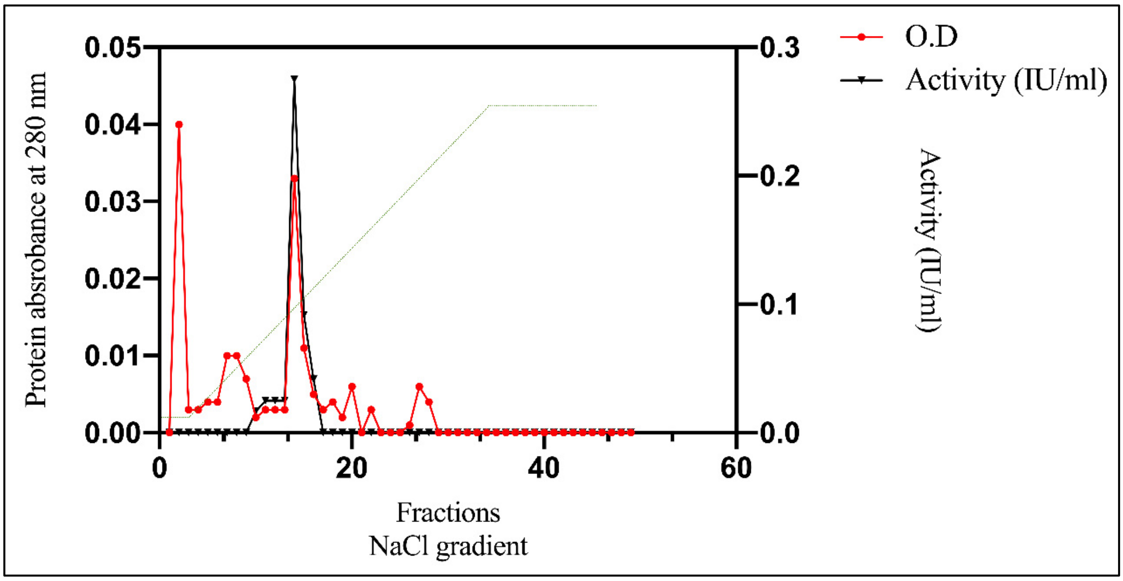

Figure S2. Elution profile of asparaginase from *L. theobromae* from the Q-FF ion exchange column. The column was pre-equilibred with 50 mM Tris buffer (pH 7) and fractions were eluted with a gradient solution of NaCl (0-1M) prepared in same buffer.

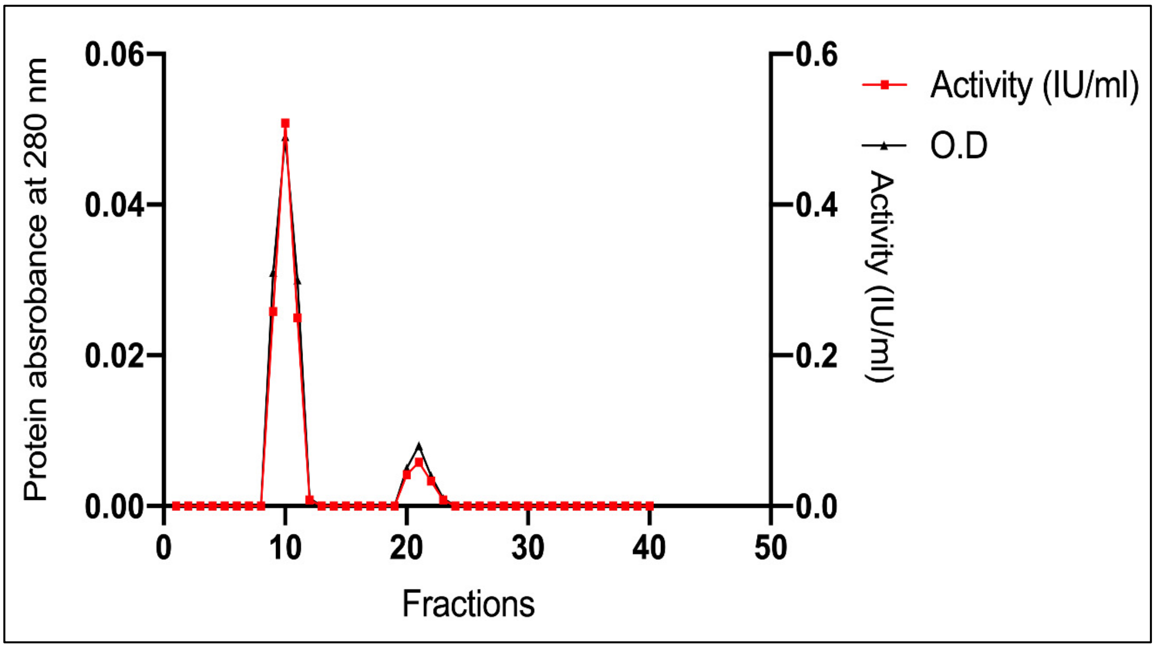

Figure S3. Elution profile of asparaginase from *L. theobromae* from the gel-filtration column (Sephadex G-100). Fractions were eluted with 50 mM Tris buffer (pH 7).
